# Allicin Improves Intestinal Epithelial Barrier Function and Prevents LPS-Induced Barrier Damages of Intestinal Epithelial Cell Monolayers

**DOI:** 10.3389/fimmu.2022.847861

**Published:** 2022-02-04

**Authors:** Jingxia Gao, Guanzhong Song, Haibo Shen, Yiming Wu, Chongqi Zhao, Zhuo Zhang, Qian Jiang, Xilong Li, Xiaokang Ma, Bie Tan, Yulong Yin

**Affiliations:** ^1^ Animal Nutritional Genome and Germplasm Innovation Research Center, College of Animal Science and Technology, Hunan Agricultural University, Changsha, China; ^2^ Key Laboratory of Feed Biotechnology of Ministry of Agriculture and Rural Affairs, Feed Research Institute, Chinese Academy of Agricultural Sciences, Beijing, China; ^3^ Laboratory of Animal Nutritional Physiology and Metabolic Process, Institute of Subtropical Agriculture, Chinese Academy of Sciences, Changsha, China

**Keywords:** allicin, intestinal barrier, IPEC-J2, oxidative damage, Nrf2, lipopolysaccharide

## Abstract

Gut barrier disruption is the initial pathogenesis of various diseases. We previously reported that dietary allicin improves tight junction proteins in the endoplasmic reticulum stressed jejunum. However, whether the allicin benefits the gut barrier within mycotoxin or endotoxin exposure is unknown. In the present study, IPEC-J2 cell monolayers within or without deoxynivalenol (DON) or lipopolysaccharide (LPS) challenges were employed to investigate the effects of allicin on intestinal barrier function and explore the potential mechanisms. Results clarified that allicin at 2 μg/mL increased the viability, whereas the allicin higher than 10 μg/mL lowered the viability of IPEC-J2 cells *via* inhibiting cell proliferation. Besides, allicin increased trans-epithelial electric resistance (TEER), decreased paracellular permeability, and enhanced ZO-1 integrity of the IPEC-J2 cell monolayers. Finally, allicin supplementation prevented the LPS-induced barrier damages *via* activating Nrf2/HO-1 pathway-dependent antioxidant system. In conclusion, the present study strongly confirmed allicin as an effective nutrient to improve intestinal barrier function and prevent bacterial endotoxin-induced barrier damages.

## Introduction

The gut epithelial barrier is composed of a single layer of enterocytes located in the inner wall of the intestine and the tight junctions between the enterocytes. Destruction of the intestinal barrier increases intestinal permeability and accelerates the translocation of pathogens as well as other harmful substances to the bloodstream (1), which are the initial pathogenesis of various diseases, such as cardiovascular and neurodegenerative diseases (2, 3). Therefore, maintaining the integrity of the intestinal barrier is a key target to prevent the development of these diseases. Intestinal epithelial cells are conjugated by tight junctional complexes, which regulate the permeability of adjacent cells and maintain the integrity of the epithelial barrier (4). The main tight junction proteins include Occludin, Claudins, and ZO-1, which are critical in regulating intestinal permeability and the epithelial paracellular leakage pathway (5).

Allicin, a sulfur-containing compound, is a kind of natural compound that can be extracted from garlic. Many studies have demonstrated its beneficial roles in anti-microbial, anti-inflammatory, anti-oxidative, and anti-cancer activities (6, 7). In addition, we previously reported that allicin, as an effective organic osmotic substance, plays an important role in regulating cells’ adaptation to endoplasmic reticulum stress (8). Attributed to the activities in inhibiting cell proliferation (9), allicin might be effective in cancer treatment (10). Besides, allicin attenuates chronic social defeat stress-induced depression which may be attributed to the regulatory roles of allicin in the gut-blood-brain axis (11). Importantly, evidence suggests that allicin benefits intestinal development in piglets (12) and large yellow croakers (13). However, the specific effects and mechanisms of allicin involved in the intestinal barrier function remain elusive.

Various adverse factors may induce intestinal barrier damage, thus initiating disease development. For example, lipopolysaccharide (LPS), cell walls from Gram-negative bacteria in the contaminated food, could disrupt the intestinal barrier in a dose-dependent manner, accompanied by pathological changes of intestinal permeability, the tight junction proteins, and oxidative stress (14, 15). Deoxynivalenol (DON), a secondary metabolite from the fungi in the corns, could easily damage the intestinal barrier (16, 17). The human colon cancer cell line Caco-2 grown in the transwell plates was commonly used as an intestinal barrier model to mimic the gut barrier of humans (18–20). However, this Caco-2 cell monolayer mimicked gut barrier has been questioned due to its characters of rapid growth and malignant proliferation. In contrast, a well-differentiated IPEC-J2 cell monolayer grown in the transwell plates can well mimic the normal intestinal barrier function (21, 22). Therefore, in the present study, IPEC-J2 cell monolayers within or without DON or LPS challenges were applied to investigate the effects and mechanisms of allicin involved in the intestinal barrier function.

## Materials and Methods

### Materials

Allicin (Cat. No. HY-N0315, purity > 97%) was purchased from MCE (Shanghai, China). Lipopolysaccharides from Salmonella enterica serotype (Cat. No. L4641), DON (Cat. No. SML1664), and fluorescein isothiocyanate dextran (FD-4, Cat. No. 46944, molar mass: 4,000) were purchased from Merck (Sigma Aldrich, Shanghai, China). ML385 (Cat. No. M8692) was kindly provided by AbMole (Shanghai, China). Phosphate buffer solution (PBS, Cat. No. 20012027), Dulbecco’s Modified Eagle’s Medium/Ham’s F-12 (DMEM/F12) cell culture medium (Cat. No. 11320082), 0.25% trypsin–EDTA–phenol red (Cat. No. 25200056), penicillin-streptomycin–glutamine (Cat. No. 10378016), and fetal bovine serum (Cat. No. 16140071) were purchased from Thermo Fisher Scientific (Shanghai, China). Six-well transwell plates (0.4-μm pore size, 24-mm diameter, Cat. No. 3491), 96-well, and 6-well cell culture plates were purchased from Corning (Shanghai, China). BeyoGold 96-well black opaque plates (Cat. No. FCP966-80pcs) were purchased from Beyotime (Shanghai, China).

### Cytotoxicity Determination of Allicin on IPEC-J2 Cells

To explore the possible cytotoxicity of allicin to IPEC-J2 cells, cells with 70% confluence in the 96-well plates were treated with a series of concentrations of allicin. Cell counting kit-8 (Cat. No. CA1210, Solarbio, Beijing, China) was used to determine the relative viability of the cells in the 96-well plates following the manufacturer’s instruction and previous published studies (23, 24).

### EdU Staining for Cell Proliferation Determination

To evaluate the possible effects of allicin on the proliferation of IPEC-J2 cells, EdU staining was performed using a Click-iT™ EdU imaging kit (Cat. No. MA0424-1, Meilunbio, Dalian, China) according to the manufacturer’s protocol and a previous published study (25). Briefly, IPEC-J2 cells were fixed with 4% paraformaldehyde for 20 min and then permeabilized with 0.5% TritonX-100 in PBS for 20 min. The pre-treated cells were incubated with a Click-iTTM reaction cocktail containing reaction buffer, Alexa Fluor^®^ 594 Azide, and reaction buffer additive for 30 min followed by 30-min incubation with Hoechst 33342.

### Maintenance of IPEC-J2 Monolayer in Transwell Plates

IPEC-J2 cells between 20th to 30th passages were seeded on the apical compartment of 6-well transwell plates at a density of 1×10^5^ cells/well. Cells were cultured with DMEM/F12 medium supplemented with 10% FBS, and 1× penicillin-streptomycin-glutamine. The culture medium was changed every two days before cell confluency and changed every day once the cells reached 100% confluency/differentiation. Cell monolayers that displayed a stable trans-epithelial electrical resistance (TEER) were used in the formal experiments.

### Barrier Damages Induced by DON or LPS

To induce barrier damage with optimal concentrations of DON and LPS, the dose-effects of DON and LPS on the cell viability IPEC-J2 monolayer were explored. A series of concentrations of DON (0, 2, 4, 6, and 8 μg/mL), and LPS (0, 1, 2, 5, 10, and 20 μg/mL) were applied for 24 h, respectively. Finally, the DON at 2 μg/mL and the LPS at 5 μg/mL were applied to induce intestinal barrier damage in IPEC-J2 monolayers. For the DON-involving experiments, the IPEC-J2 monolayers with or without 2 μg/mL allicin pretreatment were challenged with 2 μg/mL of DON for 48 h. For the LPS-involving experiments, the IPEC-J2 monolayers with or without 2 μg/mL allicin pretreatment were challenged with 5 μg/mL LPS for 48 h.

### Barrier Integrity Determination *via* TEER Measurement

The procedure for TEER measurement was described in our previous study (26). Briefly, after equilibrating the cells with PBS for 10 min at room temperature, an electrical resistance monitor (ESR-2, Millipore, USA) was used for TEER measurement. TEER values of the cell monolayers in transwell plates were calculated using readout values multiply by the basal area (4.52 cm^2^) of the apical inserts. The final calculated TEER values were normalized to the control group and used as an indicator reflecting the barrier integrity of IPEC-J2 monolayers. The equation for the TEER normalization is “Normalized TEER = (TEER values in the experimental group – TEER values of the blank control)/(average TEER values in the control group – TEER values of the blank control)”.

### Paracellular Permeability Detection

To quantify the paracellular permeability of the cell monolayer, a final concentration of 1 mg/mL FD-4 was added to the apical compartment of transwell plates. The culture medium in the basolateral compartment was taken after 30 min’s incubation. Then, the medium was slightly mixed and transferred into 96-well black opaque plates (100 μL in each well, 3 replicates). A multimode microplate reader (Infinite M Plex, Tecan, Austria) was used for detecting the fluorescent intensity (excitation, 485 nm; emission, 528 nm). The measured fluorescent intensity was normalized to the control group and used as an indicator reflecting the paracellular permeability of the IPEC-J2 monolayer. The equation for the FD-4 permeability normalization is “Normalized FD-4 permeability = fluorescent intensity in the experimental group/average fluorescent intensity in the control group”.

### Electric Cell-Substrate Impedance Sensing (ECIS) Assays and Discrete Wavelet Transform for the Barrier Function Determination

A commercial ECIS system (1600R, Applied Biophysics, Troy, NY, USA) was employed for resistance measurement to indicate the barrier function of IPEC-J2 cell monolayers. In the present study, an 8W10E-type sensing chip consisting of 8 separate wells was used. The detecting electrodes were equilibrated with 200 μL 10 mM L-cysteine for 15 min at room temperature followed by washing with 200 μL sterilized ddH_2_O twice. Subsequently, a 200 μL pre-warmed culture medium with 10% FBS was added to each well and equilibrated for 4 h at 37°C. The culture medium was removed before seeding 1.5 × 10^5^ cells in a final volume of 400 μL. The continual data were obtained within the scanning frequency of 4 kHz. To quantify the micromotion of IPEC-J2 cells, discrete wavelet transform (DWT) was introduced to refine the raw data collected from the ECIS system. A DWT-based analysis using the Daubechies 4 (dB4) mother wavelet was employed to describe resistance time series as described by a previous study (27). The cellular standard deviation (SD), variance (VAR), signal magnitude area (SMA), and Cellular-Power of the detail coefficients at level 1 were calculated by Equations [1–4] and defined as Detail-SD, Detail-VAR, Detail-SMA, and Cellular-Power, respectively.


[1] $$Cellular-SD=\sqrt{\frac{1}{n-1}\sum_{t=1}^{n}{\left(D_{j}\left(t\right)-\bar{D_{j}}\right)}^{2}}$$

[2] $$Cellular-VAR=\frac{1}{n-1}\sum_{t=1}^{n}{\left(D_{j}\left(t\right)-\bar{D_{j}}\right)}^{2}$$

[3] $$Cellular-SMA=\frac{1}{T}\sum_{t=1}^{n}\left|D_{j}\left(t\right)\right|$$

[4] $$Cellular-Power=\sqrt{\frac{1}{n}\sum_{t=1}^{n}D_{j}^{2}\left(t\right)}$$

Where *D_j_(t)* is the detail coefficients, and the n represents the length of detailed signal *D_j_(t)*.

### Immunofluorescence for Assessing Tight Junction Protein Integrity

IPEC-J2 cells (1 × 10^4^ cells/well) were seeded in a 96-well culture plate for two days. After the specific treatments, the cells were fixed with 4% paraformaldehyde for 20 min and then permeabilized with 0.2% TritonX-100 in PBS for 20 min. The cells were blocked with 5% goat serum (Cat. No. C0265, Beyotime, China) and then incubated with a 1:50 diluted ZO-1 polyclonal antibody (Cat. No. 61-7300, Thermo Fisher Scientific) overnight at 4°C. An Alexa fluor-488 secondary antibody with 1:500 dilution (Cat. No. A-11034, Thermo Fisher Scientific) was added to the cells and incubated for 1 h. Rinsed cells were sealed with an antifade solution (Cat. No. P0126, Beyotime, China). Images were captured by a fluorescence microscope (Axio Vert A1, Zeiss, Germany).

### LDH Leakage and IL-8 Secretion Determination

IPEC-J2 cells (1 × 10^5^ cells/well) were seeded in 6-well culture plates for two days. After the specific treatments, the culture medium was collected for the determinations. The procedures for LDH leakage determination were referred to a previous published study (28) and the protocol of the LDH assay kit (Cat. No. A020-2-2) purchased from Nanjing Jiancheng Bioengineering Institute. The procedures for IL-8 determination were referred to our previous study (26) and the protocol of the ELISA kit (Cat. No. CSB-E06787p) purchased from Cusabio. Results from the experimental group were normalized to the protein concentration of each sample and presented as relative percentages to the control group.

### Determination for the Activity of Mitochondrial Dehydrogenase

IPEC-J2 cells (1 × 10^4^ cells/well) were seeded in 96-well culture plates for two days. After the specific treatments, 10 μL of pre-mixed WST-8 (0.5 mM) and methoxy-PMS (20 μM) were incubated with the cells for 2h, OD_450nm_ values read from a multimode microplate reader (Infinite M Plex, Tecan, Austria) was used for assessing mitochondrial dehydrogenase activity in the cells. Results were normalized to the percentages of the control group.

### Intracellular ROS Detection

IPEC-J2 cells (1 × 10^4^ cells/well) were seeded in a 96-well culture plate for two days. After the specific treatments, the detection was carried out by referring to a protocol in reactive oxygen species (ROS) detection kit (Cat. No. S0033S, Beyotime, Shanghai, China). The ROS-tracked cells were observed with a fluorescence microscope (Axio Vert A1, Zeiss, Germany). For the quantification analysis, ROS-positive IPEC-J2 cells in BeyoGold 96-well black opaque plates were used for the fluorescence detection using an excitation wavelength of 488 nm and emission wavelength of 425 nm with a multimode microplate reader (Infinite M Plex, Tecan, Austria).

### Anti-Oxidative Parameters Detection

IPEC-J2 cells (1 × 10^5^ cells/well) were seeded in 6-well culture plates for two days. After the specific treatments, the cells were 3 times washed with pre-cooled PBS and then collected by a cell scraper. After 10 min’s ultrasonic treatment, the cell lysis was used for the tests. The detection was carried out by referring to the protocol in the kits (cell MDA assay kit/A003-4-1, SOD assay kit/A001-3-1, GSH assay kit/A061-1-1, T-AOC assay kit/A015-2-1) purchased from Nanjing Jiancheng Bioengineering Institute. Results from the experimental group were normalized to the protein concentration of each sample and presented as relative percentages to the control group.

### RNA Extraction and Real-Time PCR

The procedures for RNA extraction and Real-time PCR were described in our previous study (23). The reverse transcription kit (Evo M-MLV RT Kit, Cat. No. AG11705) was purchased from Accurate Biology (Hunan, China). The primers for RT-PCR in the present study were designed with the Primer-Blast tool based on the published cDNA sequence in the Gene Bank. GAPDH was used as the internal reference gene to determine the relative expression of targeted genes. Information on the detected genes and primers is shown in **Table S1** in supporting information.

### Protein Extraction and Western Blot

The procedures for protein extraction and Western blot were described in our previous study (8). In the present study, relative protein levels of p-Nr2 and HO-1 of the IPEC-J2 cell monolayers in each group were determined, using β-actin as an internal-reference protein.

### Statistical Analysis

Data were presented as means ± standard deviations (SD). Statistical analysis was performed using GraphPad Prism 8.0 software (CA, USA). Differences between these treatments were evaluated using one-way ANOVA. Significance difference was considered when “*P* < 0.05”.

## Results

### Effects of Allicin on the Cell Proliferation and Cell Viability of IPEC-J2 Cells

**Figure 1:**
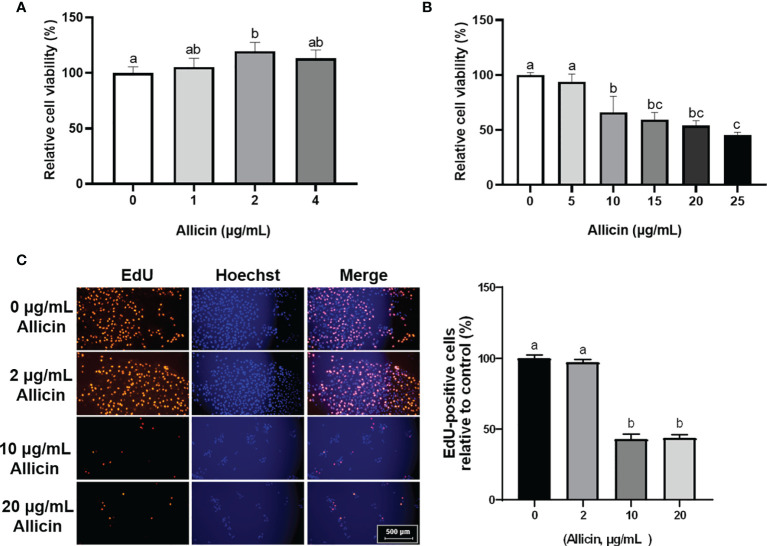
Relative viability and EdU-positive ratio of allicin supplemented IPEC-J2 cells. **(A)** Relative viability of IPEC-J2 cells treated with serial concentrations (0, 1, 2, and 4 μg/mL) of allicin. **(B)** Relative viability of IPEC-J2 cells treated with serial concentrations (0, 5, 10, 15, 20, and 25 μg/mL) of allicin. **(C)** The representative images and statistical analysis of EdU-positive IPEC-J2 cells with 0-20 μg/mL allicin supplementation. Values were presented as means ± SD, n = 3. Shared superscript letters indicate no significant difference (*P* > 0.05).

To determine the dose-effect of allicin on IPEC-J2 cells, cells of 80% confluence were incubated with allicin (0-25 μg/mL). The results (**Figures 1A, B**) demonstrated that allicin lower than 4 μg/mL was not toxic to the viability of IPEC-J2 cells, whereas concentrations higher than 10 μg/mL significantly decreased the cell viability. EdU analysis further indicated that the ratio of the EdU-positive cells in the 10 and 20 μg/mL allicin supplemented groups were significantly lower than that in the 2 μg/mL allicin supplemented and control group (**Figures 1C**).

### Effects of Allicin on the Barrier Function of DON or LPS Challenged IPEC-J2 Cell Monolayers

**Figure 2:**
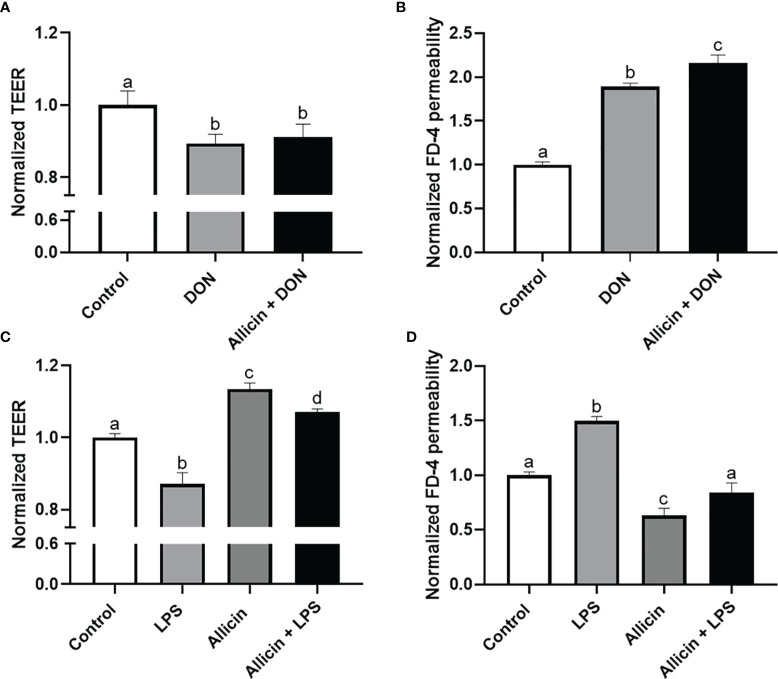
Normalized TEER and FD-4 permeability of non-treated (Control), DON-challenged (DON), LPS-challenged (LPS), allicin-treated (Allicin), allicin then DON-challenged (Allicin + DON), and allicin then LPS (Allicin + LPS) challenged IPEC-J2 cells. **(A)** Normalized TEER of IPEC-J2 monolayers challenged with or without 48 h’s DON exposure. **(B)** Normalized FD-4 permeability to IPEC-J2 monolayers challenged with or 48 h’s DON exposure. **(C)** Normalized TEER of IPEC-J2 monolayers challenged with or without 48 h’s LPS exposure. **(D)** Normalized FD-4 permeability to IPEC-J2 monolayers challenged with or without 48 h’s LPS exposure. TEER, transepithelial electric resistance; FD-4, fluorescein isothiocyanate dextran-4kDa. Values were presented as means ± SD, n = 3. Shared superscript letters indicate no significant difference (*P* > 0.05).

To choose a proper concentration of DON or LPS that damages the intestinal barrier of IPEC-J2 cells, dosage effects of DON or LPS on cell viability were determined (**Figure S1** in **Supplementary Material**). Minimum effective concentrations, which are 5 μg/mL LPS and 2 μg/mL DON, were applied to induce intestinal barrier damages in the IPEC-J2 cell monolayers. The effects of DON, LPS, and allicin on TEER values and FD-4 permeability are shown in **Figure 2**. It was indicated that the DON and LPS both decreased the TEER values and increased FD-4 permeability of the IPEC-J2 cell monolayer. Sole allicin supplementation increased the TEER values and decreased the FD-4 permeability. The allicin aggravated the DON-induced changes of TEER values and FD-4 permeability. However, the allicin significantly prevented the LPS-induced changes of TEER values and FD-4 permeability.

### Commercial ECIS Analysis for the Barrier Function of the Non-Treated, LPS-Challenged, or Allicin Then LPS-Challenged IPEC-J2 Cell Monolayers

**Figure 3:**
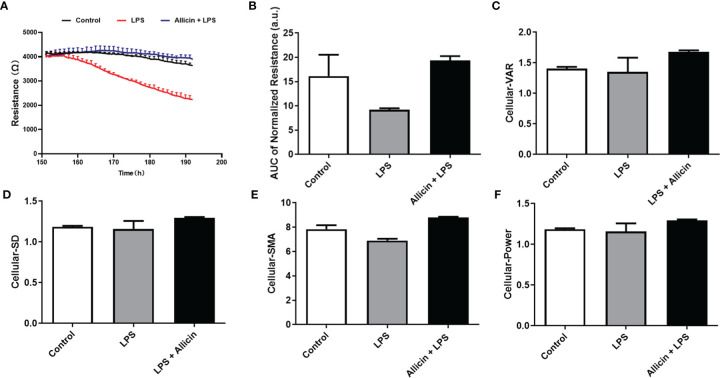
Barrier function and cellular viability of the non-treated (Control), LPS challenged (LPS), or allicin then LPS challenged (Allicin + LPS) IPEC-J2 cells monitored by ECIS. **(A)** Continual resistance values of the cell monolayers were shown. **(B)** AUC of the normalized resistance of the cell monolayers. **(C–F)** Cellular-VAR, Cellular-SD, Cellular-SMA, and Cellular-Power of the cell monolayers were shown, respectively. AUC, the area under curves; SD, standard deviation; VAR, variance; SMA, signal magnitude area. Two cell monolayer replicates for each group were used for continuous data acquisition in the ECIS system.

To validate the beneficial effects of allicin on LPS-challenged IPEC-J2 cells, commercial ECIS equipment was applied to real-time monitor the barrier function. As shown in **Figure 3A**, LPS exposure significantly decreased the resistance of the IPEC-J2 monolayers in a time-dependent manner and allicin pretreatment reversed the LPS-induced changes of resistance values. Quantified analysis of the area under curves confirmed that allicin numerically reversed LPS-induced barrier damage (**Figure 3B**). As shown in **Figures 3C–F**, allicin also numerically reversed LPS-induced changes of Standard Deviation (SD), Variance (VAR), and Signal Magnitude Area (SMA).

### Effects of Allicin on Tight Junction Integrity of LPS-Challenged IPEC-J2 Cells

**Figure 4:**
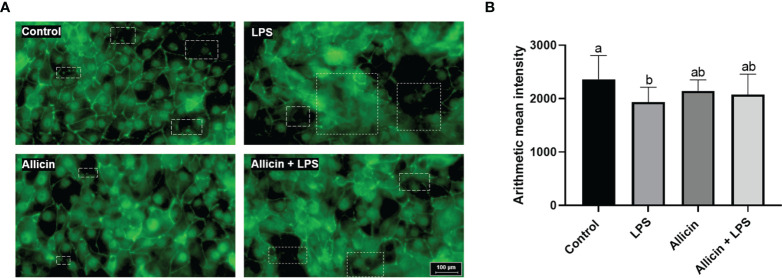
Immunofluorescent of ZO-1 protein of the non-treated (Control), LPS challenged (LPS), or allicin then LPS challenged (Allicin + LPS) IPEC-J2 cells. **(A)** Representative immunofluorescent images of ZO-1 protein were shown. **(B)** The arithmetic intensity of the images in each group was shown. Values were presented as means ± SD, n = 3. Shared superscript letters indicate no significant difference (P > 0.05).

To investigate the effects of allicin on tight junction integrity of LPS-challenged IPEC-J2 cells, ZO-1 protein was stained by immunofluorescence. As shown in **Figure 4A**, LPS damaged the ZO-1 integrity, and allicin treatment maintained better integrity of ZO-1 protein in LPS-challenged IPEC-J2 cell monolayers. However, the fluorescence intensity of the ZO-1 protein was decreased by the LPS challenges, but not totally reversed by the allicin treatment (**Figure 4B**).

### Effects of Allicin on Cell Membrane Integrity, Mitochondrial Dehydrogenase, and Proliferation of LPS-Challenged IPEC-J2 Cells

**Figure 5:**
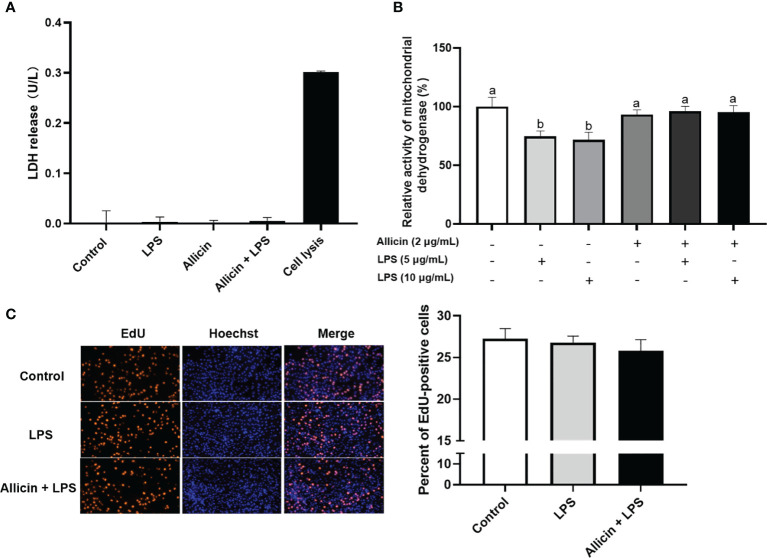
Cell membrane integrity, mitochondrial dehydrogenase, and proliferation of the non-treated (Control), LPS challenged (LPS), or allicin then LPS challenged (Allicin + LPS) IPEC-J2 cells. **(A)** The LDH release in each group was shown. **(B)** The relative activity of mitochondrial dehydrogenase was shown. **(C)** The representative images and statistical analysis of EdU-positive IPEC-J2 cells in each group were shown. LDH, lactate dehydrogenase; EdU, 5-ethynyl-2’-deoxyuridine. Values were presented as means ± SD, n = 3. Shared superscript letters indicate no significant difference (*P* > 0.05).

Based on the above observations, we are interested in whether allicin also affects the membrane integrity or proliferation of LPS-challenged IPEC-J2 cells. Specifically, the ratio of EdU-positive cells and lactate dehydrogenase (LDH) leakage to the culture medium were determined to indicate cell membrane integrity and cell proliferation. The LDH in the culture medium from each group was not detectable (**Figure 5A**). As shown in **Figure 5B**, LPS at 5 μg/mL or 10 μg/mL significantly downregulated the reducibility of mitochondrial dehydrogenase, and allicin supplementation reversed this downregulation. The LPS at 5 μg/mL and co-treatments of LPS (5 μg/mL) and allicin (2 μg/mL) did not affect the EdU-positive ratio of IPEC-J2 cells (**Figure 5C**).

### Effects of Allicin on ROS Levels and Antioxidative Parameters in LPS-Challenged IPEC-J2 Cells

**Figure 6:**
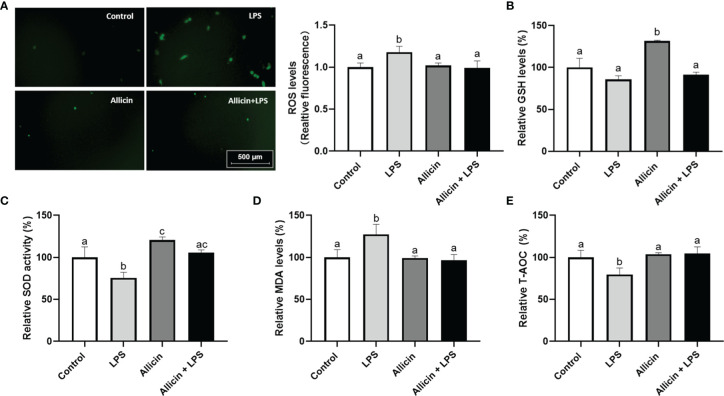
ROS levels and anti-oxidative parameters of the non-treated (Control), LPS challenged (LPS), allicin treated (Allicin), or allicin then LPS challenged (Allicin + LPS) IPEC-J2 cells. **(A)** The representative images and statistical analysis of ROS-positive IPEC-J2 cells in each group were shown. **(B)** The relative GSH levels were shown. **(C)** The relative SOD activity in each group was shown. **(D)** The relative MDA levels in each group were shown. **(E)** The relative T-AOC in each group were shown. ROS, reactive oxygen species; GSH, glutathione; SOD, superoxide dismutase; MDA, malondialdehyde; T-AOC, total antioxidant capacity. Values were presented as means ± SD, n = 3. Shared superscript letters indicate no significant difference (*P* > 0.05).

Based on the observation that allicin reversed the activities of mitochondrial dehydrogenase of IPEC-J2 challenged IPEC-J2 cells, it is surmised that allicin may be beneficial to the antioxidant system of IPEC-J2 cells. To confirm this hypothesis, we further determined ROS levels and antioxidative parameters in LPS-challenged IPEC-J2 cells by using reactive oxygen probe tracking and ELISA. As shown in **Figure 6A**, ROS levels in IPEC-J2 cells were triggered by LPS, which can be successfully pacified by allicin pretreatment. The intracellular GSH level, SOD activity, MDA levels, and T-AOC were shown in **Figures 6B–E**, respectively. Compared with the control, the solo allicin treatment improved the GSH level and SOD activity but did not affect the MDA level and T-AOC. The downregulations of SOD activity and T-AOC by LPS were reversed by allicin pretreatment. The MDA level increased by LPS was also pacified by allicin.

### Allicin Regulated the Nrf2/HO-1 Pathway in a Dose-Dependent Manner

**Figure 7:**
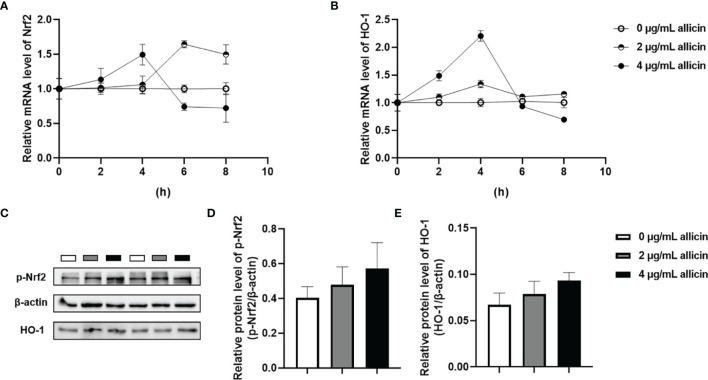
Relative levels of Nrf2 and HO-1 in the allicin pretreated IPEC-J2 cells. **(A)** The relative mRNA levels of Nrf2 during 0-8 h were shown. **(B)** The relative mRNA levels of HO-1 during 0-8 h were shown. **(C)** The presentive images of p-Nrf2 and HO-1 proteins at 8h were shown. **(D)** Statistical analysis of the relative protein levels of p-Nrf2 at 8h were shown. **(E)** Statistical analysis of the relative protein levels of HO-1 at 8h were shown. HO-1, heme oxygenase-1; Nrf2, nuclear factor erythroid 2-related factor 2. GAPDH was used as an internal reference for each target gene. Beta-actin was used as an internal reference for each target protein. Values were presented as means ± SD, n = 3.

The Nrf2/HO-1 pathway act as a key mediator for the ROS accumulation in cells. To explore whether the Nrf2/HO-1 pathway participates in allicin preventing LPS-induced intestinal barrier damages, we determined the relative mRNA levels of Nrf2, HO-1 in the IPEC-J2 cells that were pretreated with series concentrations of allicin (0, 2, and 4 μg/mL). As shown in **Figures 7A, B**, during 0-4 h treatment, allicin ranging from 0-4 μg/mL improved relative mRNA levels of Nrf2 and HO-1 in both dose-dependent and time-dependent manners. At 8 h treatment, the mRNA levels of Nrf-2 in and HO-1 in the 2 μg/mL allicin supplemented cells were significantly higher than that in the control group. However, 4 μg/mL allicin treatment for 8 h induced significantly lower mRNA levels of Nrf2 and HO-1. As shown in **Figures 7C–E**, at 8 h allicin treatment, the allicin numerically increased the phospho-Nrf2 and HO-1 in a dose-dependent manner.

### Nrf2 Inhibitor ML385 Abolished the Beneficial Roles of Allicin in LPS-Challenged IPEC-J2 Cell Monolayers

**Figure 8:**
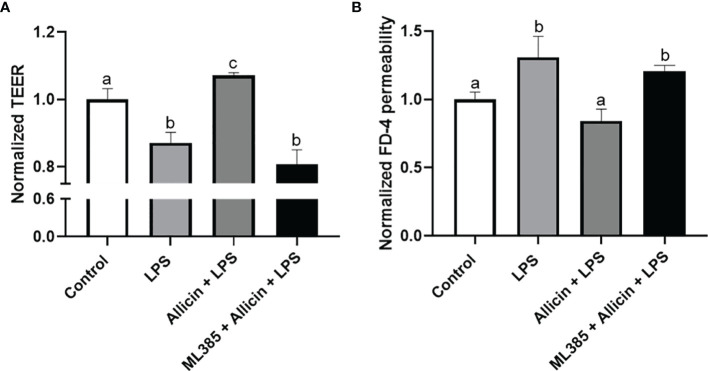
The effects of ML385 on the barrier function of IPEC-J2 cell monolayers with allicin pretreatment and LPS exposure. **(A)** Normalized TEER of IPEC-J2 monolayers. **(B)** Normalized FD-4 permeability to IPEC-J2 monolayers. Values were presented as means ± SD, n = 4. Shared superscript letters indicate no significant difference (*P* > 0.05).

To clarify whether the Nrf-2 pathway is involved in the protective roles of allicin in LPS-induced damages of IPEC-J2 cell monolayers. We pretreated the IPEC-J2 cell monolayers with Nrf2 inhibitor ML385 before the allicin pretreatment and LPS exposure. Noteworthy, the ML385 at 5.0 μg/mL successfully reversed the protective roles of allicin in LPS-challenged IPEC-J2 cell monolayers (**Figures 8A, B**).

## Discussion

Various chronic diseases have been correlated with the malfunction and irreversible oxidative damage of the gut barrier (29). In terms of mechanism, a leaky gut-blood barrier results in increased intestinal permeability to bacterial endotoxin and adverse factors in the gut, which induces various inflammatory diseases. Thus, the strategy for preventing gut barrier damage is an acknowledged way to avoid related diseases such as cardiovascular and neurodegenerative diseases (29, 30). Results in the present study strongly confirmed that allicin is a natural compound of antioxidants that prevents the LPS-induced barrier damages, possibly *via* enhancing the Nrf2/HO-1 associated pathway.

Allicin is a kind of natural compound that can be extracted from garlic. Many studies have demonstrated its beneficial roles of anti-microbial, anti-inflammatory, and anti-oxidative properties. Importantly, evidence suggests that allicin benefits intestinal development in piglets (12) and large yellow croakers (13). We demonstrated that allicin at concentrations below 10 μg/mL was not toxic to the IPEC-J2 cells, and 2 μg/mL allicin increased cell viability to the cells. In the present study, the WST-8 method (functional components in the CCK-8 kit) was employed to assess the viability of the cells. WST-8 for cell viability detection principally reflects the total reducibility of mitochondrial dehydrogenase in the cells. As indicated by the results, the low concentrations of allicin (2 μg/mL) indeed increased the anti-oxidative parameters of the IPEC-J2 cells, implying better reducibility of the treated cells. Thus, we surmised the increased cell viability here might be attributed to the increased reducibility of the allicin-supplemented cells. Besides, the high dose of allicin damages the cell viability of IPEC-J2 cells, which is consistent with a recent study which reported that allicin inhibits the proliferation of human epidural scar fibroblasts in a dose-dependent manner (31). Therefore, the high concentration allicin-induced cell viability damage might be attributed to the proliferation inhibition and cell cycle re-distribution (9). In consideration of the low dosage garlic dietary intake as a seasoning, the safety concentration of 2 μg/mL allicin was applied for the barrier function experiments on IPEC-J2 cell monolayers. We firstly investigated the protective effects of allicin on intestinal epithelial barrier damages induced by DON, as the DON-contaminated food could easily disrupt gut barrier function, as indicated by our previous study (23). DON was revealed to inhibit cell proliferation in different conditions (32, 33). In the present study, the allicin both inhibited cell proliferation and aggravated the DON-induced barrier damages within the experimental condition, which may be attributed to the synergy-effects of DON and allicin in restraining DNA formation (34, 35). Contrary to the roles of allicin in the DON-induced damage model, allicin successfully prevented the LPS-induced damages to intestinal barrier function, which prompts us to further validate its effects and explore potential mechanisms.

Typically, the TEER as an indicator of paracellular resistance has been applied for intestinal epithelial cells *in vitro* models (23, 36, 37). However, TEER measurement is time-consuming, and cell stress is triggered at room temperature which makes it impossible to pursue real-time and continual detection. To validate the protective roles of allicin in LPS-induced barrier damage, we applied a commercial ECIS system to real-time detect the intestinal barrier function of IPEC-J2 monolayers. Notably, ECIS also confirmed allicin reversed the adverse changes induced by LPS stimulation. However, how allicin improves intestinal function and prevents LPS-induced barrier damage is not clear and needed to be furtherly explored.

Previous evidence has indicated that LPS can trigger inflammatory cytokines (38) and destroy the cell membrane integrity (39) of intestinal epithelial cells *in vitro*. In the present study, neither the LPS nor allicin induced LDH leakage from the cells, which indicates LPS only damaged the intracellular junctions instead of the cell membrane itself. In line with previous studies (40, 41), we indeed observed LPS improved the mRNA level of IL-8 in IPEC-J2 cells, and numerically enhanced the IL-8 secretion to the culture medium (**Figure S2** in **Supplementary Material**). Noteworthy, the allicin supplementation furtherly enhanced the mRNA level in LPS-challenged IPEC-J2 cells and aggravated the IL-8 secretion (**Figure S2** in **Supplementary Material**). Generally, the initiative up-regulation of inflammatory cytokines *in vivo* is supposed to activate the phagocytosis of immune cells for clearing the pathogens or endotoxin invasion (42, 43). The allicin-induced inflammatory cytokine upregulation may be attributed to the initiative immune activation. However, the allicin failed to pacify the LPS-induced inflammatory response in the IPEC-J2 cell monolayers due to a deficiency of immune cell co-incubation.

Interestingly, we observed a significant decrease in the reducibility of mitochondrial dehydrogenase in the LPS-challenged IPEC-J2 cells, and this decrease was reversed by the allicin pretreatment. This result prompted us to explore whether allicin exerts its protective role on the barrier function *via* anti-oxidative ways. The intracellular reactive oxygen species (ROS), glutathione (GSH), superoxide dismutase (SOD), malondialdehyde (MDA), and total antioxidant capacity (T-AOC) are parameters to assess redox homeostasis, in which the Nrf2/HO-1 pathway plays key regulatory roles (44–46). In the present study, we noticed that the allicin prevented the LPS-induced oxidative stress, which may be considered as a key mechanism that participates in preventing barrier damage of the IPEC-J2 cell monolayers. Previous studies reported that allicin mitigates hepatic injury (47), hepatotoxicity (48), and cardiac hypertrophy (49) *via* activating Nrf-2 pathways. In the present study, allicin ranging from safe concentrations was verified to regulate the Nrf2 and HO-1 mRNA levels both in dose-dependent and time-dependent manners. Therefore, it is surmised that the Nrf-2 pathway might be involved in the protective roles of allicin in LPS-induced damages of IPEC-J2 cell monolayers. To verify this hypothesis, we pretreated the IPEC-J2 cell monolayers with Nrf2 inhibitor ML385 before the allicin pretreatment and LPS exposure. Noteworthy, Nrf2 inhibitor ML385 successfully abolished the beneficial roles of allicin in LPS-challenged IPEC-J2 cell monolayers. Thus, it is implied that allicin prevented LPS-induced barrier damages *via* enhancing the Nrf2/HO-1 associated antioxidant system.

## Conclusion

In summary, the present study demonstrated the protective roles of allicin in LPS challenged intestinal epithelial barrier. In terms of mechanism, the Nrf2/HO-1 pathway activation and the antioxidant system were verified to be involved in the beneficial roles of allicin in maintaining barrier integrity and preventing LPS-induced oxidative damages. Thus, it is proposed that allicin is an effective nutrient to regulate intestinal barrier function and prevent bacterial endotoxin-induced barrier damages. Further *in vivo* studies are warranted to validate the roles of the Nrf2/HO-1 pathway and allicin involved in the Gram-negative bacteria-associated intestinal barrier damages.

## Data Availability Statement

The original contributions presented in the study are included in the article/**Supplementary Material**. Further inquiries can be directed to the corresponding author.

## Authors Contributions

JG: Conceptualization, Methodology, and Writing an original draft. GS: Investigation, and Writing-review-editing. HS: Investigation, and Writing-review-editing. YW: Software and Validation. CZ: Software and Validation. ZZ: Software and Validation. QJ: Conceptualization, Funding Acquisition, Resources, Supervision, and Writing-review-editing. XL: Review-editing. XM: Formal analysis. BT: Review-editing. YY: Review-editing. All authors contributed to the article and approved the submitted version.

## Funding

This work was supported by grants from the “Shen Nong Scholar Funding of Hunan Agricultural University”, the “Changsha Municipal Natural Science Foundation (Grant No. kq2014068)”, the “Hunan Natural Science Foundation (Grant No. 2021JJ40233)”, the “Science and Technology Innovation Program of Hunan Province (Grant No. 2021RC3090)”, the “Open Project Program of Key Laboratory of Feed Biotechnology, the Ministry of Agriculture and Rural Affairs of the People’s Republic of China”, and the “Earmarked Fund for China Agriculture Research System (CARS-35)”.

## Conflict of Interest

The authors declare that the research was conducted in the absence of any commercial or financial relationships that could be construed as a potential conflict of interest.

## Publisher’s Note

All claims expressed in this article are solely those of the authors and do not necessarily represent those of their affiliated organizations, or those of the publisher, the editors and the reviewers. Any product that may be evaluated in this article, or claim that may be made by its manufacturer, is not guaranteed or endorsed by the publisher.
