# Supplementary material for: Allicin Improves Intestinal Epithelial Barrier Function and Prevents LPS-Induced Barrier Damages of Intestinal Epithelial Cell Monolayers
Source: Front Immunol. 2022 Feb 4;13:847861. doi: 10.3389/fimmu.2022.847861 (PMC8854216; doi:10.3389/fimmu.2022.847861)
Supplement: Supplementary file 1 [file DataSheet_1.docx]

**Supplementary Material for:**

# Allicin improves intestinal epithelial barrier function and prevents LPS-induced barrier damages of intestinal epithelial cell monolayers

Jingxia Gao^1, †^, Guanzhong Song^1, †^, Haibo Shen^1, †^, Yiming Wu^1^, Chongqi Zhao^1^, Zhuo Zhang^1^, Xiaokang Ma^1^, Qian Jiang^1, 2, *^, Xilong Li^2^, Bie Tan^1^, Yulong Yin^1, 3^

^1^ Animal Nutritional Genome and Germplasm Innovation Research Center, College of Animal Science and Technology, Hunan Agricultural University, Changsha, Hunan 410128, China

^2^ Key Laboratory of Feed Biotechnology of Ministry of Agriculture and Rural Affairs, Feed Research Institute, Chinese Academy of Agricultural Sciences, Beijing 100081, China

^3^ Laboratory of Animal Nutritional Physiology and Metabolic Process, Institute of Subtropical Agriculture, Chinese Academy of Sciences, Changsha, Hunan 410125, China

† Contributed equally to this work

* Correspondence should be addressed to Qian Jiang ([jiangqian@hunau.edu.cn](mailto:jiangqian@hunau.edu.cn))

**Figure S1.** Dosage effects of LPS and DON on cell viability of IPEC-J2 cells. (A) Relative viability of IPEC-J2 cells treated with serial concentrations (0, 1, 2, 5, 10, and 20 μg/mL) of LPS. (B) Relative viability of IPEC-J2 cells treated with serial concentrations (0, 2, 4, 6, and 8 μg/mL) of DON. Values were presented as means ± SD, n = 3. Shared superscript letters indicate no significant difference (*P* > 0.05).

**Figure S2.** Effect of allicin and LPS on the mRNA levels and secretion of IL-8. (A) Relative mRNA levels of IL-8 in IPEC-J2 cells were shown. (B) Relative IL-8 secretion by IPEC-J2 cells was shown. GAPDH was used as an internal reference for each target gene. Values were presented as means ± SD, n = 3. Shared superscript letters indicate no significant difference (*P* > 0.05).

**Table S1.** Primers in the present study.

| **Gene** | **Primer Sequences（5’-3’）** | **Length（bp）** | **Access No.** |
| --- | --- | --- | --- |
| GAPDH | F: GGGCATGAACCATGAGAAGT  R: AAGCAGGGATGATGTTCTGG | 230 | XM_019925987.2 |
| IL-8 | F: AGTTTTCCTGCTTTCTGCAGCT  R: TGGCATCGAAGTTCTGCACT | 104 | NM_001004027 |
| HO-1 | F: AGCTGTTTCTGAGCCTCCAA  R: CAAGACGGAAACACGAGACA | 130 | XM_005671981 |
| Nrf-2 | F: CACCACCTCAGGGTAATA  R: GCGGCTTGAATGTTTGTC | 125 | XM-005671981 |

**Notes：**glyceraldehyde-3-phosphate dehydrogenase (GAPDH), Interleukin-8 (IL-8), heme oxygenase-1 (HO-1), nuclear factor erythroid 2-related factor 2 (Nrf-2).
